# Aptamer-CRISPR/Cas12a-Based Lateral Flow Technique for Visualized Rapid Detection of Endogenous Damage Factor Neu5Gc in Red Meat

**DOI:** 10.3390/foods14162879

**Published:** 2025-08-19

**Authors:** Yuxi Guo, Honglin Ren, Han Wang, Xuepeng Duan, Shuaihao Qi, Xi Yang, Chunyi Shangguan, Haosong Li, Yansong Li, Pan Hu, Qiang Lu, Shiying Lu

**Affiliations:** State Key Laboratory for Diagnosis and Treatment of Severe Zoonotic Infectious Diseases, Key Laboratory for Zoonosis Research of the Ministry of Education, Institute of Zoonosis, College of Veterinary Medicine, Jilin University, Changchun 130062, China; yxguo21@mails.jlu.edu.cn (Y.G.); renhl@jlu.edu.cn (H.R.); hanw21@mails.jlu.edu.cn (H.W.); duanxp24@mails.jlu.edu.cn (X.D.); qish24@mails.jlu.edu.cn (S.Q.); qyang23@mails.jlu.edu.cn (X.Y.); sgcy23@mails.jlu.edu.cn (C.S.); lhs948892860@outlook.com (H.L.); l_ys92305@163.com (Y.L.); hupan84@163.com (P.H.); qlu@jlu.edu.cn (Q.L.)

**Keywords:** aptamer truncation, trans-cleavage, dual-labeled probe, food safety biomarker, signal amplification, colorimetric detection

## Abstract

The N-glycolylneuraminic acid (Neu5Gc), a major salivary acid molecule found on the cell surface of animals such as pigs, cows, and sheep, can be metabolically incorporated into the body through consumption of animal-derived foods like red meat. This leads to an immune response and chronic inflammation in individuals who do not naturally produce Neu5Gc, including humans and poultry, further increasing the risk of cancer. The trans-cleavage activity of Cas12a is activated by the recognition of the target aptamer by the crRNA, resulting in the cleavage of the dual-labeled probe. By combining this with immunochromatographic techniques, we established a chromatographic test strip assay that allows immediate on-site detection of Neu5Gc contamination in non-red meat samples devoid of Neu5Gc. Further optimization enabled specific detection within 25 min with a minimum detectable limit of 10 ng/mL. These analyses successfully detected the spiked samples and actual samples containing Neu5Gc. The developed lateral flow test strips based on aptamer-Cas12a can be utilized for detecting Neu5Gc contamination in non-red meat food products, animal bioproducts, and poultry feeds.

## 1. Introduction

N-Glycolylneuraminic acid (Neu5Gc) is a sialic acid variant characterized by a pyranose ring structure and a nine-carbon composition. It is prevalent in the non-neural tissues and bodily fluids of vertebrates and echinoderms but is notably absent from healthy human tissues [1]. In humans, a premature truncation of the reading frame, caused by the deletion of an exon in the CMAH gene, leads to the inactivation of CMP-Neu5Gc hydroxylase. This inactivation results in the organism’s inability to synthesize Neu5Gc [2]. Despite the absence of endogenous synthesis of Neu5Gc in humans, it can be ingested through the consumption of Neu5Gc-rich foods [3] such as red meat [4] and milk [5]. This exogenous Neu5Gc can accumulate in the body, triggering an antigen-antibody response that may lead to chronic inflammation. Excessive consumption of beef and lamb has been linked to the development of various chronic non-communicable diseases, including type 2 diabetes [6], non-alcoholic fatty liver disease (NAFLD) [7], cardiovascular disease [8], and cancer [9]. The prevalence of chronic diseases is increasing, thereby posing a significant threat to public health and well-being. A plethora of studies have been conducted in 10 European countries, Sweden, and Australia to investigate the relationship between red meat consumption and colorectal cancer. The majority of the extant research indicates a positive correlation between the consumption of red meat and the risk of developing colorectal cancer [10,11]. Breast cancer constitutes 25% of all female cancer diagnoses on a global scale and is also associated with the consumption of red meat. A comparison of datasets reveals that individuals who consume substantial quantities of red meat are predisposed to an 8% elevated risk of developing breast cancer, in contrast to those who consume minimal amounts of red meat. Research indicates that postmenopausal women who consume red meat have a 9% higher risk of developing breast cancer [12]. In relation to the matter of stomach cancer, Bonequi et al. [13] conducted a study on the association between diet and stomach cancer in Latin American populations. This study demonstrated a significant association between high red meat consumption and the risk of stomach cancer. Consequently, excessive or prolonged consumption of red meat could increase the risk of cardiovascular disease and cancer. Currently, no international food safety authority, including the U.S. FDA [14] or EFSA [15], has defined a Maximum Residual Limit (MRL) for N-glycolylneuraminic acid (Neu5Gc) in food products [16]. However, increasing scientific and clinical evidence has linked Neu5Gc to chronic inflammation, atherosclerosis, and certain cancers due to its accumulation in human tissues from red meat consumption. These health concerns underscore the importance of sensitive and rapid detection methods for Neu5Gc in food.

Presently, Neu5Gc detection primarily depends on liquid-phase instruments, which necessitate expensive equipment, specialized procedures, and sample derivatization, therefore restricting its widespread application. Immunodetection employs Western blot techniques using mono- or polyclonal IgY antibodies against gangliosides GM2 and GM3 [17], along with immunohistochemistry for identifying glycolipid-containing Neu5Gc in specific tumor tissues [18]. However, the chicken IgY polyclonal antibodies utilized exhibit limitations such as low specificity and heightened background reactivity. Consequently, there is a pressing demand for the development of innovative detection probes and assays to accommodate the associated research requirements. Aptamers, often referred to as “chemical antibodies”, are obtained through Systematic Evolution of Ligands by Exponential Enrichment (SELEX) technology [19]. They can address the challenge of preparing murine antibodies to Neu5Gc due to its animal-derived nature, which hampers the development of immunological detection techniques. By using aptamers as probes, a novel, rapid, sensitive, and practical detection method can be developed. This can be achieved by integrating immunological detection technology with CRISPR gene technology [20]. This approach offers more advantages compared to traditional high-performance liquid chromatography-mass spectrometry (HPLC-MS) [21] and antibody-based detection technologies. It can be utilized to detect any products, tissues, and biologics derived from red meat, serum, and milk, as well as related tumors.

Based on the truncated 55 bp aptamer as the target sequence, a Neu5Gc aptamer-Cas12a lateral flow test strip assay was established. This assay leverages the precise recognition of the Cas12a enzyme and the rapid visualization capabilities of lateral flow chromatography test strips. The optimal conditions for this system were determined to be a double-labeled probe concentration of 5 μM, an aptamer content of 5 μL, a reaction temperature of 37 °C, and a reaction time of 15 min. The specificity of the assay was high, and the sensitivity was excellent, with a minimum detection limit of up to 10 ng/mL. The assay performed well when applied to spiked samples and actual samples. This assay was used to analyze Neu5Gc contamination in non-red meat samples (e.g., white meat such as poultry meat and aquatic products), poultry feed, and animal biological products that do not contain Neu5Gc. This represents a significant advancement in the detection of Neu5Gc, offering a rapid, sensitive, and practical method for monitoring Neu5Gc levels in a variety of contexts.

## 2. Materials and Methods

### 2.1. Materials

The nucleic acid sequences utilized in this study, as presented in Table 1, were synthesized and purified via high-performance liquid chromatography (HPLC) by Sangon Biotech (Shanghai, China). Neu5Gc and KDN (2-Keto-3-deoxy-D-glycero-D-galacto-nononic acid, a Neu5Gc analog used for specificity testing) were obtained from Cosun Technology Co., (Shanghai, China). The Neu5Ac (N-Acetylneuraminic Acid) was obtained from TCI Co., Ltd. (Shanghai, China). N-acetyl-D-mannosamine (Sigma-Aldrich, St. Louis, MI, USA, #A7225); D-(+)-glucose, sucrose, and D-(+)-maltose monohydrate were obtained from Si-nopharm Chemical Reagent Corporation (Shanghai, China). The crRNA was designed based on the specific aptamer N2A9.55 sequence. DEPC-treated water: Purchased from Thermo Fisher, Waltham, MA, USA (Cat. #AM9935), used to avoid RNase contamination during crRNA handling. The LbCas12a (Lachnospiraceae bacterium Cas12a enzyme, used in the CRISPR assay), dual-labeled probes, and CRISPR/Cas12a-specific nucleic acid test strips were all purchased from Bio-lifesci (Guangzhou, China). The dual-labeled single-stranded DNA probe (5′-biotin-TCCCCCCCCT-FAM-3′), hereafter referred to as Bio-reporter-FAM, where ‘Bio’ stands for biotin and ‘FAM’ is carboxyfluorescein. The test strips are assembled in the following layers: sample pad, conjugate pad (embedded colloidal gold particles labeled with anti-FAM antibody), nitrocellulose membrane (with T line coated with goat anti-murine antibodies and C line coated with streptavidin), absorbent pad, and backing card. 10× Buffer: 200 mM Tris-HCl, 500 mM NaCl, 50 mM MgCl_2_, pH 7.5. Main instruments and their sources are PCR amplification during SELEX: Thermo Fisher Veriti™ Thermal Cycler (Waltham, MA, USA); OD measurements: BioTek ELx800 Microplate Reader (Burlington, VT, USA); HPLC purification of aptamers: Agilent 1260 Infinity HPLC System (Santa Clara, CA, USA).

### 2.2. Aptamer Truncation Optimization

In the previous stage of the research group [22], a random oligonucleotide ssDNA library with a total length of 81 nucleotides was constructed using Primer Premier 5.0. The middle part contained 40 random base sequences, while the two ends had fixed sequences consisting of 21 and 20 nucleotides, respectively. With Neu5Gc as the screening target, 15 rounds of screening were conducted using the ssDNA library immobilized magnetic bead-SELEX technology. The homology of the sequences was analyzed using DNAMAN 9.0 software (https://www.dnaman.net/ (acessed on 1 May 2022)), and the secondary structure of the sequences was predicted through m-fold (https://www.unafold.org (accessed on 20 May 2022)), resulting in four candidate aptamers. The affinity, pH stability, and specificity of the candidate aptamers were evaluated by the Enzyme-Linked Immunosorbent Assay (ELISA) method, and the best aptamer, N2A9, was obtained. Based on the aptamer’s ELISA quantification, a method for detecting Neu5Gc was established. The Neu5Gc-BSA conjugate was coated at the bottom of the microplate, and the excess sites on the microplate were blocked with a blocking agent before adding the samples and the aptamer N2A9. The immobilized Neu5Gc would compete with the Neu5Gc in the samples for the limited nucleic acid aptamer; after the competition, the mixed solution in the microplate was washed away, and the aptamer N2A9 that bound to Neu5Gc-BSA was located at the bottom of the microplate. The biotin-labeled aptamer N2A9 specifically bound to SA-HRP (Streptavidin Conjugated with Horseradish Peroxidase), introducing the enzyme molecules into the system; finally, HRP catalyzed the substrate TMB (3,3’,5,5’-Tetramethylbenzidine) to produce a color reaction. The target substance was quantitatively detected based on the change in the absorbance OD_450_ (optical density: used to indicate the degree to which the detected object absorbs light) value of the colored substrate. The absorbance (OD_450_) value of each well was measured using an enzyme-labeled instrument, and the higher the OD_450_ value, the lower the content of Neu5Gc in the sample. The antibody Neu5Gc-BSA was diluted to a concentration of 1 μg/mL, and 5% skimmed milk was used for 2 h of blocking at 37 °C. The optimal working concentration of the aptamer was 50 nmol/L. The incubation conditions were 37 °C for 1 h. The secondary antibody SA-HRP was incubated at 37 °C for 1 h. The optimal working time of the TMB substrate was 10 min. The standard curve equation was y = −0.3417x + 0.837 (R^2^ = 0.9927), with a detection limit of 0.71 ng/mL.

The stem-loop structure formed by certain bases on the aptamer may be the primary region responsible for its binding to the target. Roceky [23] et al. proposed that a scientifically sound method for cutting nucleic acid aptamers should be based on their secondary structure. The two fixed primer regions were removed in a sequential manner, and the sequence of the random region was analyzed as a clipped nucleic acid aptamer. The resulting sequence of the clipped nucleic acid aptamer is presented in Table 1. In accordance with the prediction of the binding site for molecular docking, the nucleotides of the same secondary structural unit in the vicinity of the binding site were retained, and the redundant nucleotides and other secondary structural units were deleted. The truncated sequence was synthesized, and the binding effect of the truncated sequence to the target was determined by indirect competition ELISA [22]. In comparison with the binding effect of the previous full-length aptamer, the truncated sequence was demonstrated to be effective if the OD value increased or remained relatively stable.

### 2.3. Aptamer Optimization Selection and crRNA Design

**Figure 1:**
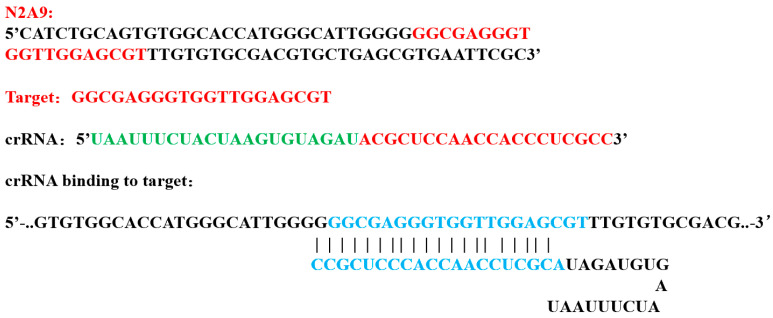
Design of crRNA. For the aptamer N2A9 sequence, using the Benchling platform, high-quality crRNAs complementary to it were designed. The red is target sequence; the green is scaffold sequence; the blue is the binding part of the aptamer target sequence and the crRNA target sequence.

A high-quality crRNA was designed based on the aptamer N2A9 sequence as a target using the Benchling platform (Figure 1). In accordance with the aptamer truncation design following the selected aptamer N2A9 (55 bp) and the untruncated aptamer N2A9, the objective is to verify the feasibility of the selection and obtain favorable results for subsequent experiments. The procedure is as follows: First, 5 μL of 100 nM aptamer is added to the bottom of the Eppendorf tube (Ep tube), which is then equilibrated to room temperature. Subsequently, the CRISPR/Cas12a reaction is conducted. The reaction system was as follows: The solution was prepared with 5 μL of 10× Buffer, 5 μL of crRNA, 5 μL of Dual-labeled probes, 5 μL of Cas12a, and 10 μL of DEPC-treated water. The CRISPR system was added to the cap of the EP tube, which was then subjected to an immediate centrifugation step and incubated for 15 min at 37 °C in a dark environment. Following the incubation period, the EP tube was opened, and the test strip binding pad end (arrow end) was inserted into the reaction tube, ensuring that the liquid level did not exceed the maximum line.

### 2.4. Construction of Aptamer-Cas12a Test Strip System and Validation of Design Principles

**Figure 2:**
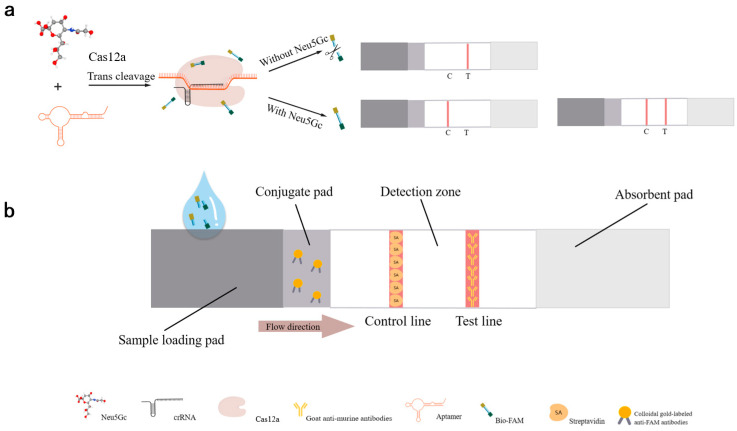
Schematic diagram of a Neu5Gc detection test strip based on Cas12a of aptamer. (**a**): Detection principle of the Neu5Gc lateral flow test strip based on aptamer-Cas12a. (**b**): Working principle of the test strip. “Target molecule” refers to Neu5Gc; “Target sequence” refers to the aptamer N2A9.55 recognized by the crRNA; “Reporter” refers to the dual-labeled ssDNA probe; Bio’ stands for biotin, and ‘FAM’ is 6-carboxyfluorescein.

The CRISPR/Cas system enables Cas12a to accurately target the target sequence and confer non-specific cleavage activity (cleaving the Bio-reporter-FAM ssDNA as collateral cleavage activity of the CRISPR/Cas12a system). Using the Neu5Gc-targeting aptamer as the target sequence and combining Cas12a with the lateral flow test strip, a detection platform based on aptamer-CRISPR/Cas12a is established for the detection of Neu5Gc. The detection principle is as shown in Figure 2: with the highly specific and high-affinity aptamer N2A9.55bp as the reaction target sequence, the crRNA of the Cas12a system is designed and synthesized. The test strip combines the pad with colloidal gold particles labeled with anti-FAM antibody, the T line is coated with mouse secondary antibody, and the C line is coated with streptavidin. Through the recognition of the target sequence by the crRNA, the Cas12a system’s trans-activation of the cutting activity is activated, cutting the double-labeled probe into two parts. The probes at both ends are modified with FAM and biotin. The part with the FAM label combines with the gold particles labeled with FAM antibody on the pad to form a FAM-FAM antibody-gold particle complex, which is eluted to the T line coated with goat anti-murine antibodies and then binds to the mouse secondary antibody, resulting in color development. The judgment results are as follows.

When there is no Neu5Gc in the sample to be tested, the aptamer is in a free state and can be recognized and bound by the crRNA, thereby activating the trans-cutting activity of the Cas12a system, cutting the double-labeled probe into two parts, and the part with the FAM label combines with the gold particles labeled with FAM antibody on the pad to form a FAM-FAM antibody-gold particle complex, which is eluted to the T line coated with goat anti-murine antibodies and then binds to the goat anti-murine antibodies, resulting in color development. At this time, since there is no Neu5Gc in the sample, the test result is negative;

When the sample to be tested contains Neu5Gc, because the aptamer binds to Neu5Gc and cannot be recognized and bound by the crRNA, it cannot activate the trans-cutting activity of the Cas12a system. The double-labeled probe remains intact and combines with the gold particles labeled with FAM antibody on the pad to form a gold particle-FAM antibody-FAM-Bio complex, which can be developed on either the T line coated with goat anti-murine antibodies or the C line coated with streptavidin. The color development depends on the amount of Neu5Gc in the sample. If the content is high, there is no free aptamer, and only the C line develops color; if the content is low, both the C line and the T line develop color, because the sample contains the Neu5Gc. The test result is positive.

To prevent contamination and streamline the process, a one-step approach was employed to integrate the Cas12a cleavage reaction and the test strip reaction within a single reaction vessel. This involved a total of 50 µL of the complete aptamer-Cas12a test strip, as illustrated in Table 2. In summary, the aptamer was initially bound to the target Neu5Gc at room temperature for 10 min at the bottom of the EP tubes. Subsequently, the CRISPR/Cas12a detection system, which was embedded in the cap, was briefly removed to allow full contact with the target and incubated for 15 min at 37 °C in the light-protected zone. Subsequently, the Cas12a detection system embedded in the cap of the tube was briefly transiently separated, allowing for full contact between the aptamer and target, and incubated for 15 min at 37 °C, protected from light. Test strips were then inserted into the EP tubes for reaction, to be read for the area to develop color.

### 2.5. Aptamer-Cas12a Test Strip System Optimization

In order to optimize the detection of Neu5Gc, it is essential to consider key factors such as aptamer content, probe concentration, CRISPR/Cas12a cutting time, and temperature.

(1) In order to achieve the optimal detection performance of the aptamer-Cas12a test strips while minimizing costs, two key parameters, aptamer content and probe concentration, were optimized. The aptamer content was set to 1 μL, 2 μL, 5 μL, and 10 μL without modifying other conditions to ensure that the probe was sufficiently cut while maintaining sufficient binding efficiency. The probe concentration was optimized by setting it to 1 μM, 5 μM, and 10 μM to control the variables of the reaction. The CRISPR/Cas reaction was conducted in the same reaction system, and the results were read by inserting the test strip into the EP tube.

(2) To reduce the time cost and enhance the field detection, even with optimized detection time, the CRISPR/Cas12a cutting time was set to 5, 10, 15, 30, 60, and 90 min, respectively. Subsequently, the CRISPR/Cas reaction was conducted, and finally, the test strip was inserted into the EP tube for result reading.

(3) To prevent the inactivation of the Cas12a protein under conditions of high temperature, an optimization of the reaction temperature was conducted. The reaction temperature was set to 13 °C, 21 °C, 29 °C, 37 °C, 45 °C, 53 °C, and 61 °C to identify the optimal reaction temperature. The conditions of the Neu5Gc lateral flow test strip assay for aptamer-Cas12a were optimized by optimizing the aforementioned parameters to achieve the most effective detection outcome.

### 2.6. Specificity Determination of Neu5Gc Lateral Flow Detection Test Strips Based on Aptamer-Cas12a

The system was optimized with the objective of assessing the specificity of the method. Six Neu5Gc analogues, Neu5Ac, KDN (ketodeoxynonanoic acid), N-acetyl-D-mannosamine, maltose, sucrose, and glucose, were selected, and the optimized reaction conditions and system were applied to the selected analogues. The aptamer was combined with the selected Neu5Gc analogs and incubated at room temperature for 10 min. Subsequently, the CRISPR/Cas reaction was conducted, and the results were analyzed by inserting test strips into EP tubes.

### 2.7. Aptamer-Cas12a Test Strip Sensitivity Assay

To assess the sensitivity of the method, a series of diluted Neu5Gc standards were prepared at concentrations of 5 ng/mL, 10 ng/mL, 50 ng/mL, 100 ng/mL, 500 ng/mL, and 1000 ng/mL, respectively. Subsequently, the sensitivity of the test strips for the aptamer-Cas12a assay was analyzed by incubating the aptamer with different concentrations of Neu5Gc standards at room temperature for 10 min to allow for the CRISPR/Cas reaction. Finally, the test strips were inserted into EP tubes to read the results.

### 2.8. Aptamer-Cas12a-Based Neu5Gc Lateral Flow Test Strip Spiking and Actual Sample Detection

To assess the potential applicability and authenticity of the lateralized test strip method for Neu5Gc detection, chicken was selected as the subject for verification of the test strip’s feasibility in real sample detection. The recovery of the test strip was evaluated by adding varying concentrations of Neu5Gc. Standard Neu5Gc (5 ng/mL, 100 ng/mL, and 1000 ng/mL) was added to the chicken samples as spiked samples. Subsequently, three portions of pork, three portions of beef, three portions of lamb, three portions of chicken, three portions of duck, three portions of fish, and three portions of shrimp were taken as the actual samples. The following procedures were then performed on the meat samples: extraction of Neu5Gc [16].

A 0.1 g meat sample was weighed, 0.9 mL of a 0.1 M NaOH solution was added, the sample was homogenized, and it was then incubated at 37 °C for 30 min. The sample was neutralized with concentrated hydrochloric acid, and the resulting neutralized homogenate was taken and mixed with an equal volume of 4 M acetic acid. This mixture was incubated at 80 °C for 3 h, cooled to room temperature, and then centrifuged at 14,000 rcf for 10 min. The resulting supernatant was filtered through a 0.22 μm filter membrane, thus creating the test solution.

A 5 µL aliquot of the test solution was combined with an equal volume of the 100 nM aptamer and incubated at room temperature for 10 min. This mixture was then introduced into the Cas12a reaction system for a CRISPR/Cas reaction. Finally, the test strip was inserted into the EP tube to obtain the results.

## 3. Results

### 3.1. Analysis of Truncation Optimization of Nucleic Acid Aptamers

**Figure 3:**
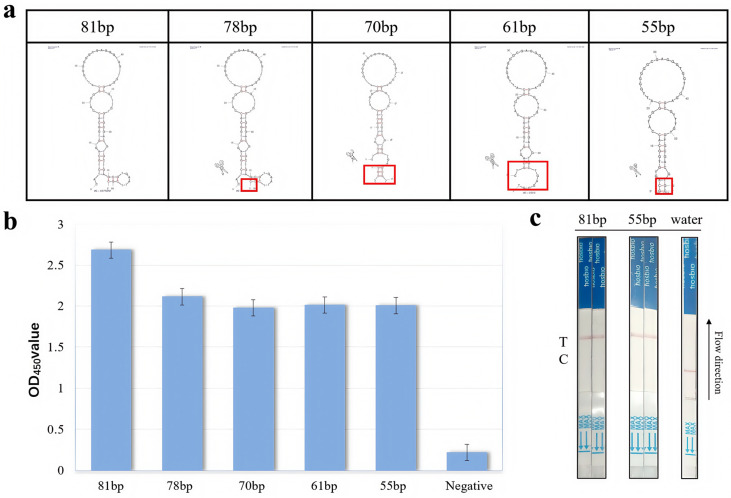
Aptamer selection results. (**a**): Secondary structure of the truncated sequence of aptamer N2A9. The red box in the picture represents the truncated sequence. (**b**): Activity analysis of the truncated sequence of aptamer N2A9. OD_450_ is that HRP–TMB reaction in ELISA assays, as the oxidized product absorbs strongly at 450 nm. (**c**): Selection of aptamers.

The full-length sequence of an aptamer typically contains redundant bases that serve only to provide unnecessary contact support. These non-essential bases do not interact with the Neu5Gc and may introduce uncertainty in the aptamer structure, thereby destabilizing the binding affinity to the target in the reaction system. The optimization of sequence truncation has the potential to markedly enhance the affinity of nucleic acid aptamers while concurrently reducing the elevated synthetic expense associated with excessively lengthy sequences. A secondary structure analysis of N2A9 reveals comparable energies and analogous specific secondary structure domains, which typically encompass stem-loop structures, G tetrasomes, bulges, or pseudoknots. These domains frequently exhibit binding capabilities with Neu5Gc. Given the stereostructural characteristics of the Neu5Gc molecule, it is plausible that the stem-loop structural domain of the N2A9 aptamer may facilitate direct binding to Neu5Gc. To investigate the specific structural domains of the nucleic acid aptamer and to obtain shortened sequences with high binding affinity, the secondary structure prediction software (mfold3.6 (https://www.unafold.org (accessed on 20 May 2022)) was employed to analyze the N2A9 aptamer, which was truncated to retain the individual loops or combinations of loops at different sites, including hairpin loops, inner loops, and multiple loops. The sequence truncation of the conserved structural stem loop of aptamer N2A9 was optimized, resulting in the production of 78 bp, 70 bp, 61 bp, and 55 bp truncated nucleic acid aptamers (Figure 3a). These were sent to BioCompany for synthesis and labeling with biotin at their 5’ end for indirect competition ELISA for binding effect analysis. As illustrated in Figure 3b, aptamer N2A9.81bp exhibited the highest OD_450_, the highest binding effect to Neu5Gc, and the highest truncation effect. Figure 3b demonstrates that the aptamer N2A9.81 bp exhibits the highest OD value and the most robust binding effect with Neu5Gc. The truncated nucleic acid aptamers 78 bp, 70 bp, 61 bp, and 55 bp also demonstrate notable binding effects, which substantiates the efficacy of the truncation approach. Upon activation of the Cas12a-crRNA complex by the aptamer, collateral cleavage of the Bio-reporter-FAM occurs, generating a red band at the T line as a visual indication of a positive result. Although FAM is a fluorescent dye, in this study, it was used as an epitope tag for recognition by anti-FAM antibodies. The OD at 450 nm was measured from ELISA plates using TMB substrate, unrelated to FAM fluorescence. These aptamers were subjected to an aptamer-Cas12a test strip reaction, the results of which are presented in Figure 3c. The 81 bp and 55 bp aptamers were cleaved, and the T line displayed a colorful reaction, indicating a positive outcome. Given the comparable affinity and the reduction in sequence, the final selection was N2A9.55bp, which was deemed the optimal choice for the construction of Neu5Gc test strips, offering a cost-effective and time-efficient synthesis process while minimizing the probability of sequence errors.

### 3.2. Proof of Principle Validation of Neu5Gc Lateral Flow Detection Test Strips Based on Aptamer-Cas12a

**Figure 4:**
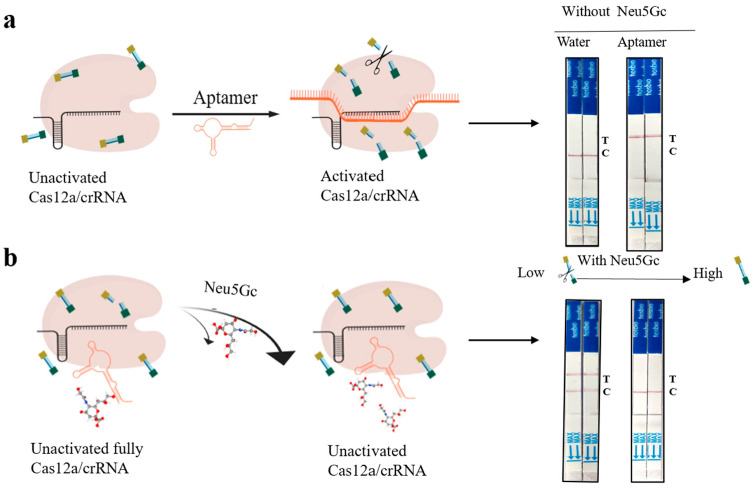
(**a**): Schematic of the CRISPR/Cas12a-based detection of Neu5Gc. (**b**): Visual results from lateral flow strips at varying Neu5Gc concentrations. Increased Neu5Gc concentration leads to reduced T-line signal due to reduced Cas12a activation.

In Figure 4, as a negative control, no Neu5Gc was added, resulting in no activation of the Cas12a/crRNA complex and thus no cleavage of the Bio-reporter-FAM probe. This was evidenced by the fact that the probe was not cut and the test strip displayed a C line. Conversely, the presence of an ssDNA aptamer activated the trans-cutting activity of Cas12a/crRNA, resulting in the probe being cut and the release of fluorescent signals. This was further evidenced by the appearance of a T line on the test strip, which proved that the CRISPR system was available for use. Subsequently, the target Neu5Gc was introduced for detection purposes. Prior to this, the aptamer and Neu5Gc were incubated for 10 min at room temperature to ensure sufficient binding affinity between the two. As the concentration of Neu5Gc increased, the trans-cutting activity of the CRISPR system exhibited a gradual decline, accompanied by a progressive enhancement in the color intensity of the C line on the test strip. Conversely, the T line demonstrated a gradual attenuation in color intensity until it became undetectable.

### 3.3. Optimization Results of Aptamer-Cas12a Test Strip Probe Concentration and Aptamer Content

**Figure 5:**
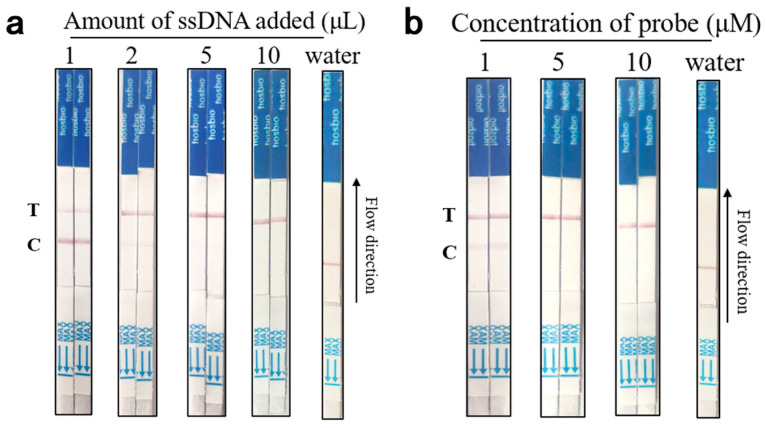
Aptamer-Cas12a test strip system optimization results. (**a**): Results of aptamer content optimization; (**b**): Results of probe concentration optimization.

In order to achieve optimal detection performance of aptamer-Cas12a assay test strips, two key parameters, aptamer content and probe concentration, were optimized. In the absence of a target, the optimization process was initiated by removing and equilibrating 100 nM aptamer N2A9.55 bp to room temperature. Subsequently, the aptamer content was varied in four increments (1 μL, 2 μL, 5 μL, and 10 μL) to ascertain the optimal concentration for the assay. Following this, the CRISPR/Cas12a reaction was conducted. The aptamer with different contents was added into the CRISPR system and incubated at 37 °C for 15 min, protected from light. Following incubation, the ep tube was opened, and the end of the binding pad of the test strip (arrow end) was inserted into the reaction tube, ensuring that the liquid level did not exceed the uppermost end of the binding pad. It was essential that the reading area be fully infiltrated and the results be read within 10 min. As illustrated in Figure 5a, when the aptamer content was 5 μL or 10 μL, only the T line exhibited coloration, thereby demonstrating optimal CRISPR system cutting activity. To minimize reagent costs, the subsequent experiments were conducted using an aptamer content of 5 μL.

Subsequently, probe concentration optimization was conducted. Various concentrations of the probe, including 1 μM, 5 μM, and 10 μM, were introduced into the reaction system, as illustrated in Figure 5b. It was observed that when the probe concentration was set to 5 μM, the system exhibited the optimal performance, with complete cutting of the probe and the presence of a colorful T line. Accordingly, the subsequent experiments were conducted using an aptamer content of 5 μL with a probe concentration of 5 μM. The optimized reaction system, exhibiting the most optimal performance, was composed of 5 μL of aptamer (100 nM), 5 μL of 10× Buffer, 5 μL of crRNA (1 μM), 5 μL of Bio-reporter-FAM (5 μM), 5 μL of Cas12a protein (1 μM), and 25 μL of DEPC-treated water.

### 3.4. Aptamer-Cas12a Test Strip Reaction Time and Temperature Optimization Results

**Figure 6:**
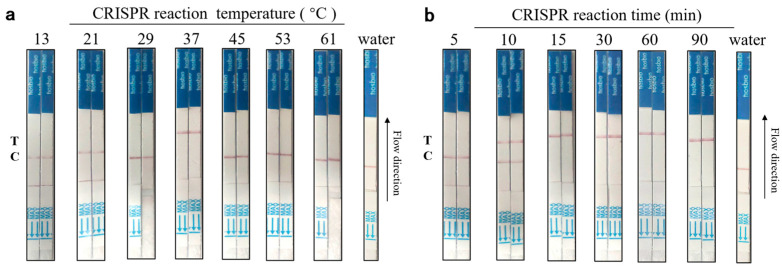
Aptamer-Cas12a test strip system optimization results. (**a**): CRISPR reaction temperature optimization results; (**b**): CRISPR reaction time optimization results.

To optimize reaction conditions, we initially excluded Neu5Gc and monitored the cleavage activity of Cas12a under varying temperature and time settings. The principal reaction parameters in the CRISPR/Cas12a-Neu5Gc assay were optimized. It was observed that the Cas12a enzyme is inactivated at temperatures exceeding a certain threshold. To circumvent this issue and ensure the optimal functioning of the enzyme at both high and low temperatures, the reaction temperatures were optimized. The optimal reaction temperatures were selected to achieve rapid and sensitive detection. A total of 5 μL of aptamer (100 nM), 5 μL of 10× Buffer, 5 μL of crRNA (1 μM), 5 μL of Bio-reporter-FAM (5 μM), 5 μL of Cas12a protein (1 μM), and 25 μL of DEPC-treated water were combined at varying temperatures (13 °C, 21 °C, 29 °C, The reaction was conducted at temperatures of 37 °C, 45 °C, 53 °C, and 61 °C for 15 min to prevent light-induced degradation. Subsequently, test strips were introduced to quantify the generated values. By optimizing the temperature, the test strips were recorded. As illustrated in Figure 6a, at a temperature of 37 °C, the T line of the test strip exhibited a color change, indicating the CRISPR system’s cutting effect. In contrast, at other temperatures, the C line of the test strip displayed a color change, suggesting that the CRISPR system did not exert a cutting effect. Therefore, the optimal reaction temperature was determined to be 37 °C.

To prevent the occurrence of erroneous test results due to prolonged reaction times, crRNA degradation, and protein inactivation, it is essential to optimize the reaction time. Therefore, it is of particular importance to select the optimal reaction time in order to achieve rapid and sensitive detection of Neu5Gc. A total of 5 μL of aptamer (100 nM), 5 μL of 10× Buffer, 5 μL of crRNA (1 μM), 5 μL of Bio-reporter-FAM (5 μM), 5 μL of Cas12a protein (1 μM), and 25 μL of DEPC-treated water were combined and incubated at 37 °C for varying periods (5 min, 10 min, 15 min, 30 min, 60 min, and 90 min) in the absence of light. Subsequently, the test strips were introduced for data acquisition. The test strips were recorded at each optimized time point. As illustrated in Figure 6b, when the reaction time is equal to or greater than 15 min, the T line of the test strips exhibits a color change, indicating that the CRISPR system is functioning as a cutting agent. Conversely, when the reaction time is less than 15 min, the C line of the test strips displays a color change, demonstrating that the CRISPR system is not performing its cutting function. The 15-min reaction time is optimal for cutting due to its suitability for immediate on-site detection, which enhances the efficiency of the detection process.

### 3.5. Based on the Specific Determination Results of the Lateral Flow Test Strip for Neu5Gc Using the Aptamer-Cas12a System

**Figure 7:**
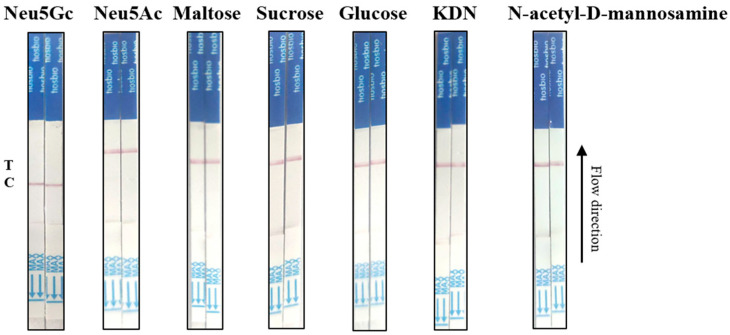
Aptamer-cas12a dipstick specificity determination. Indicates the results of the Neu5Ac, KDN, N-acetyl-D-mannosamine, maltose, sucrose, and glucose test strip at a concentration of 1 μg/mL, with Neu5Gc as a positive control.

To evaluate the specificity of the aptamer-Cas12a test strips, assays were conducted using 1000 ng/mL of Neu5Ac, KDN (ketodeoxynonanoic acid), N-acetyl-D-mannosamine, maltose, sucrose, and glucose. Firstly, the aptamer was incubated with the Neu5Gc analogues for 10 min at room temperature. Subsequently, the CRISPR/Cas reaction was performed, and finally, the test strips were inserted into EP tubes. The results are presented in Figure 7. At 1000 ng/mL Neu5Gc, the absence of a T line may be due to reduced Cas12a activation resulting from aptamer saturation by Neu5Gc, preventing crRNA binding. Given that Neu5Gc analogues do not bind specifically to the aptamer, the CRISPR system was found to exert trans-cleavage activity, resulting in the cleavage of the probe and the subsequent appearance of the T line, which exhibited a distinct coloration. This indicated that the test strip exhibited good specificity. The results demonstrate that our established method exhibits high specificity for the detection of Neu5Gc and is capable of accurately distinguishing Neu5Gc from other common analogs.

### 3.6. Aptamer-Cas12a Test Strip Sensitivity Assay Results

**Figure 8:**
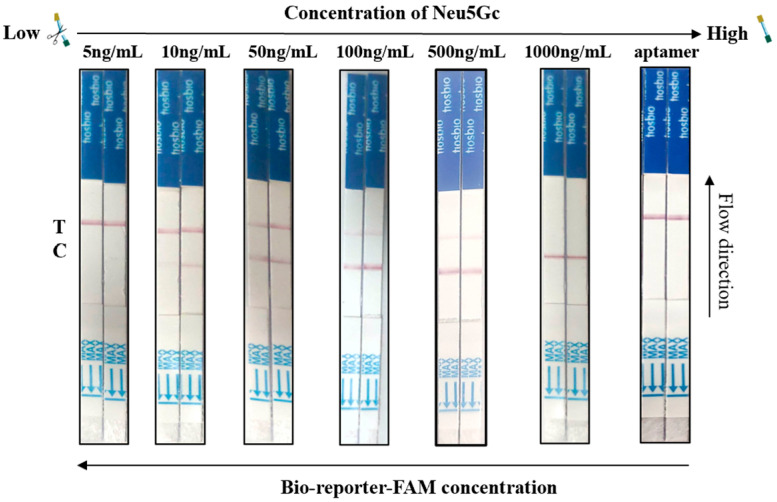
Aptamer-cas12a test strip sensitivity determination. The study employed a series of diluted Neu5Gc standards (5 ng/mL, 10 ng/mL, 50 ng/mL, 100 ng/mL, 500 ng/mL, and 1000 ng/mL) and aptamers (negative control) as samples.

The study employed a series of diluted Neu5Gc standards (5 ng/mL, 10 ng/mL, 50 ng/mL, 100 ng/mL, 500 ng/mL, and 1000 ng/mL) and aptamers (negative control) as samples for test strips. The results are presented in Figure 8. In the assay, the standard concentration of 5 ng/mL or lower was undetectable. Conversely, when the concentration of the Neu5Gc standard was 10 ng/mL or higher, the C line exhibited coloration. When Neu5Gc concentration ranged from 10 ng/mL to 500 ng/mL, both T and C lines were visible. At 1000 ng/mL, only the C line remained. As the concentration of the Neu5Gc standard increased, the color of the C line deepened, and the color of the T line became lighter. At a concentration of 1000 ng/mL, the color of the T line disappeared completely, and only the color of the C line remained. Consequently, when the concentration of Neu5Gc in the sample was below 10 ng/mL, the T line of the test strip exhibited coloration, indicating a negative result. Conversely, when the concentration of Neu5Gc in the sample exceeded 10 ng/mL, the T and C lines of the test strip displayed coloration, or solely the C line displayed coloration, signifying a positive result. The above results indicate that the sensitivity value of this aptamer-Cas12a test strip for the visual detection of Neu5Gc is 10 ng/mL.

### 3.7. Results of Neu5Gc Lateral Flow Test Strip Spiking Assay Based on Aptamer-Cas12a

**Figure 9:**
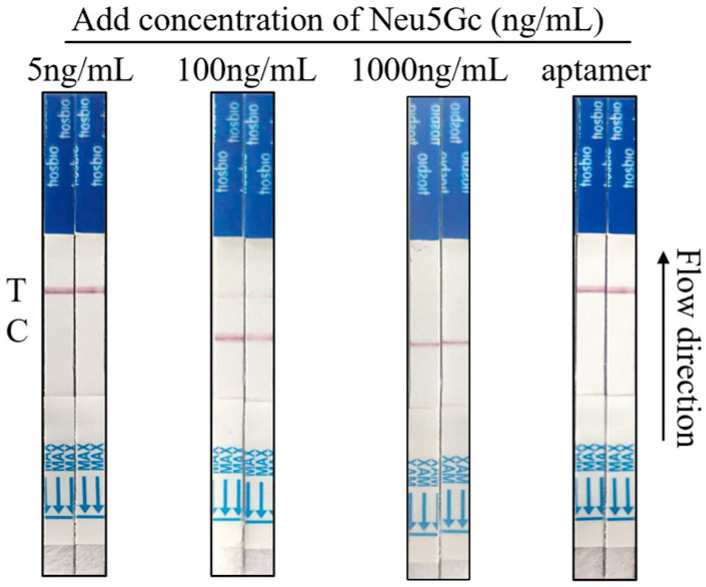
Aptamer-Cas12a test strip spiking recovery assay. The objective of this study was to determine the recovery of Neu5Gc standards at concentrations of 5 ng/mL, 100 ng/mL, and 1000 ng/mL when spiked into chicken samples.

The Neu5Gc standard concentrations of 5 ng/mL, 100 ng/mL, and 1000 ng/mL were added to chicken samples for spiking recovery, with the objective of verifying the performance of the test strip. The results are presented in Figure 9. When the spiked sample was 5 ng/mL, the CRISPR system was cut, and only the T line was observed, indicating a negative result. When the spiked sample was 100 ng/mL, both T and C lines exhibited coloration, confirming a positive result. Only the C line displayed coloration, suggesting inhibition of Cas12a activity due to excess Neu5Gc. Overall, the applicability of the test was satisfactory.

### 3.8. Actual Sample Detection of Neu5Gc Lateral Flow Test Strips Based on Aptamer-Cas12a

**Figure 10:**
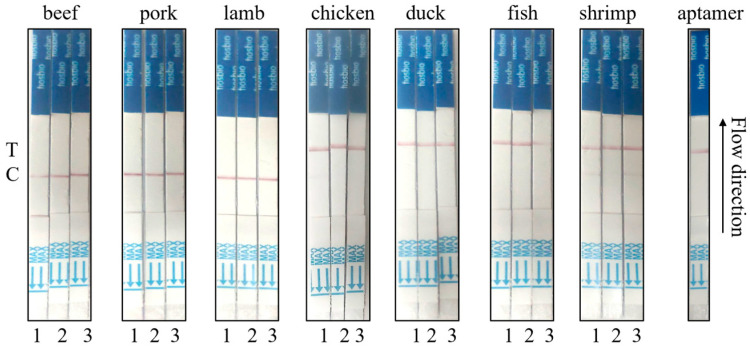
Aptamer-cas12a test strip actual sample determination. The validation process involved the analysis of actual samples, with three portions of pork, beef, and lamb and three portions of chicken, duck, fish, and shrimp meat.

Three portions each of pork, beef, and mutton, as well as three portions each of chicken, duck, fish, and shrimp meat, were procured from fresh supermarkets for the purpose of verifying the actual samples. Initially, the samples were treated in order to extract Neu5Gc in red meat, with the objective of observing the reaction of test strips with aptamer-Cas12a. As illustrated in Figure 10, all three test strips of beef, pork, and lamb samples exhibited positive results with the C line coloration. Conversely, all three test strips of chicken, duck, and fish samples demonstrated negative results with the T-line coloration. Additionally, two test strips of shrimp samples displayed positive results with both the T and C lines.

## 4. Discussion

Neu5Gc, a non-human sialic acid, has been implicated in various chronic inflammatory diseases due to its dietary accumulation from red meat. Studies have reported its association with diseases such as atherosclerosis, colorectal cancer, and systemic inflammation. Varki et al. [24] demonstrated that Neu5Gc-containing glycans could trigger an immune response leading to chronic inflammation, particularly in individuals with high red meat intake. These findings highlight the urgent need for accurate and field-deployable methods for Neu5Gc detection in meat products. The discovery of CRISPR (clustered regularly interspaced short palindromic repeats) technology as an immune system derived from prokaryotic organisms has led to its widespread use in the development of molecular diagnostics [25]. This is due to the fact that it possesses programmable and accurate gene recognition and cleavage capabilities [26]. A series of biosensing platforms for the detection of nucleic acids [27], proteins [28], small molecules [29], metal ions, and other analytes have been developed by leveraging the trans-cutting ability of Cas12a [30], Cas14a [31], and Cas13a/b [32]. In these biosensing systems, the introduction of single-stranded DNA (or RNA) signal reporter probes into the CRISPR-Cas reaction system enables the precise monitoring of the specific recognition events of DNA (or RNA) sequences by Cas nuclease. This is achieved by recording changes in specific signals, such as fluorescence and color changes [33], which provide valuable insights into the interaction between the probes and target sequences. However, the majority of these innovative CRISPR-based biosensing techniques have been developed with the use of fluorescent reporter probes, necessitating the employment of specialized optical detection devices or instruments for their detection. This presents a significant challenge to the widespread implementation of these technologies in POCT (prompt onset testing) [34].

LFT (lateral flow chromatography test strips) has been the subject of considerable interest due to its convenience and visualization and has been employed extensively in the construction of diagnostic methods for immunoassays and nucleic acid detection [35]. Nevertheless, there has been a paucity of research into the construction of biosensing platforms that couple LFT and CRISPR-Cas systems [36]. Some methods employ RPA [37], LAMP [38], or other amplification techniques in conjunction with test strips for detection. However, non-specific amplification occurs during the amplification process, and primer dimers can result in false positives due to their ability to produce results on the test strips. CRISPR/Cas12a is capable of specifically recognizing the target, thereby circumventing the false-positive effect generated by non-specific amplification and primer dimers [39]. Consequently, CRISPR/Cas12a exhibits enhanced specificity in the context of LFT test strips. The integration of the LFT platform with CRISPR-Cas technology facilitates the expeditious interpretation of results through a straightforward visual assessment, obviating the necessity for sophisticated optical instrumentation typically employed in conventional fluorescent probe-based CRISPR methodologies.

The aptamer-based combination of the CRISPR-Cas system with LFT comprises four main steps: The procedure entails four main steps: (1) extraction of Neu5Gc from meat samples; (2) incubation of the aptamer with the samples to be tested at room temperature; (3) activation of the Cas12a trans-cleavage activity to cleave the ssDNA probe; and (4) insertion of the test strip to visually visualize the strip [40]. The aptamer-CRISPR/Cas12a assay strips were optimized to be maintained at 37 °C by the portable assay device, obviating the need for repeated temperature adjustments and special instrumentation. When both Neu5Gc and crRNA are present in the sample to be analyzed, they compete with the aptamer for specific interactions. Although the affinity between Neu5Gc and the CRISPR system has not been directly measured, based on the observed signal patterns, we speculate that at high Neu5Gc concentrations, aptamer binding sites become saturated, limiting crRNA access and thereby reducing Cas12a activation. They are suitable for use in the field or low-resource laboratories. Consequently, the aptamer-CRISPR/Cas12a test strips represent a rapid, straightforward, and visually discernible technology for the prompt detection of Neu5Gc in red meat. Research has shown that red meat contains the highest levels of Neu5Gc, with beef containing the most, followed by pork, lamb, and rabbit. Varki’s [24] study of the Neu5Gc content in pork and beef found that beef contains 30.1 µg/g of Neu5Gc, compared to 25.5 µg/g in pork. The nucleic acid aptamer-based test strips and ELISA and HPLC [41] and MS [42] detection methods developed in this experiment for the endogenous pathogenic factor Neu5Gc in red meat were compared. It is evident that both the HPLC and MS instruments boast a high degree of precision, with the personnel responsible for their operation possessing extensive and highly specialized training and qualifications. The assessment of Neu5Gc-specific immune responses is hindered by three factors [43]. Firstly, the presence of a single source of antigen. Secondly, the absence of a diverse array of antigen sources. Thirdly, the elevated background reactivity. Consequently, the establishment of an effective immune response is challenging. However, the utilization of conventional laboratories is subject to certain constraints. The results of the study are presented in Table 3. A comparison of the aptamer-Cas12a test strip detection method with other methods of detection, including those related to detection time, temperature, and required instruments, clearly demonstrates the superiority of the former in terms of on-site rapid and immediate detection. Compared to the technique used by S. Gong et al. [44] to detect Neu5G using aptamers and chromatographic test strips, our method involves aptamer recognition, CRISPR/Cas12a activation, and lateral flow readout, with signal amplification achieved through cross-cleavage of dual-labeled probes. The visual detection limit is more sensitive, and the simplicity and convenience of the method make it more suitable for on-site detection. This not only enables the immediate detection of Neu5Gc in red meat in the field and significantly streamlines the operational procedure but also allows for the detection of non-red meat samples that do not contain Neu5Gc (e.g., Furthermore, the investigation of Neu5Gc contamination in white meat, such as poultry and aquatic products, poultry feeds, and animal biological products, has promising applications and potential for on-site monitoring. In conclusion, this CRISPR/Cas12a-aptamer-based colorimetric biosensor offers a rapid and visual tool for Neu5Gc detection in red meat, with potential applications in food safety and non-immunogenic meat labeling.

## 5. Conclusions

The N2A9 aptamer was truncated and optimized by removing the fixed sequences at both ends while retaining the secondary structure of the random sequence in the middle. All four truncated aptamers exhibited favorable binding activity with the Neu5Gc. Based on the 55 bp truncated aptamer, a Neu5Gc lateral flow test strip assay utilizing aptamer-Cas12a was successfully established. The reaction conditions and system were further optimized as follows: 5 μL of aptamer content, a concentration of 5 μM for the double-labeled probe, a reaction temperature of 37 °C, and a reaction time of 15 min. The assay demonstrated high sensitivity up to 10 ng/mL, excellent specificity, and reliable detection results between spiked and actual samples. Both spiked samples and actual samples were effectively detected using this simple, rapid, visualized procedure, which can be applied for on-site analysis of Neu5Gc contamination in non-red meat samples (e.g., white meat such as poultry or aquatic products), poultry feeds, and animal bioproducts devoid of Neu5Gc.

## Figures and Tables

**Table 1:** Truncated nucleic acid aptamers.

Sequence Name	Sequences (5’–3’)	Sequence Lengths
N2A9.81	CATCTGCAGTGTGGCACCATGGGCATTGGGGGGCGAGGGTGGTTGGAGCGTTTGTGTGCGACGTGCTGAGCGTGAATTCGC	81 bp
N2A9.78	CATCTGCAGTGTGGCACCATGGGCATTGGGGGGCGAGGGTGGTTGGAGCGTTTGTGTGCGACGTGCTGAGCGTGAATTCGC	78 bp
N2A9.70	CATCTGCAGTGTGGCACCATGGGCATTGGGGGGCGAGGGTGGTTGGAGCGTTTGTGTGCGACGTGCTGAGCGTGAATTCGC	70 bp
N2A9.61	CATCTGCAGTGTGGCACCATGGGCATTGGGGGGCGAGGGTGGTTGGAGCGTTTGTGTGCGACGTGCTGAGCGTGAATTCGC	61 bp
N2A9.55	CATCTGCAGTGTGGCACCATGGGCATTGGGGGGCGAGGGTGGTTGGAGCGTTTGTGTGCGACGTGCTGAGCGTGAATTCGC	55 bp
Remark	Underscores are truncated removal sequences	

**Table 2:** Reaction system of aptamer-Cas12a test strips.

Reaction	System Name	Volumetric (µL)
10 min at room temperature	Liquid to be examined	5(0)
	Aptamer100 nM	5
CRISPR/Cas12a reaction 37 °C for 15 min	Buffer	5
	LbCas12a, 1 µM	5
	crRNA, 1 µM	5
	Dual-labeled probes, 5 µM	5
	DEPC water	20(25)
	Total	50

**Table 3:** Comparison of the benefits and drawbacks of the two testing methods.

Detection Method	Detection Time	Detection Temperature	Minimum Detection Limit	Required Instrument
HPLC [41]	10 h	18–25 °C	0.02 ng/μL	High Performance Liquid Chromatography
MS [42]	12 h	18–25 °C	0.02pg/μL	Mass spectrometer
Aptamer-ELISA [22]	17 h	37 °C	0.71 ng/mL	microplate reader
Aptamer-Cas12a test strip	25 min	37 °C	10 ng/mL	Metal bath or Warm water bath

## Data Availability

The original contributions presented in this study are included in the article. Further inquiries can be directed to the corresponding author.
